# A conserved loop sequence of the proteasome system depupylase Dop regulates substrate selectivity in *Mycobacterium tuberculosis*

**DOI:** 10.1016/j.jbc.2022.102478

**Published:** 2022-09-10

**Authors:** Jin Hee Yoo, Shoshanna C. Kahne, K. Heran Darwin

**Affiliations:** Department of Microbiology, New York University School of Medicine, New York, New York, USA

**Keywords:** pupylation, depupylation, *Mycobacterium tuberculosis*, proteasome, ACN, acetonitrile, MS, mass spectrometry, PPS, Pup-proteasome system, TB, tuberculosis, TBST, Tris-buffered saline with Tween-20, TMT, tandem-mass tag

## Abstract

Mycobacteria use a proteasome system that is similar to a eukaryotic proteasome but do not use ubiquitin to target proteins for degradation. Instead, mycobacteria encode a prokaryotic ubiquitin-like protein (Pup) that posttranslationally modifies proteins to mark them for proteolysis. Pupylation occurs on lysines of targeted proteins and is catalyzed by the ligase PafA. Like ubiquitylation, pupylation can be reversed by the depupylase Dop, which shares high structural similarity with PafA. Unique to Dop near its active site is a disordered loop of approximately 40 amino acids that is highly conserved among diverse *dop*-containing bacterial genera. To understand the function of this domain, we deleted discrete sequences from the Dop loop and assessed pupylation in mutant strains of *Mycobacterium tuberculosis*. We determined that various Dop loop mutations resulted in altered pupylome profiles, in particular when mutant *dop* alleles were overexpressed. Taken together, our data suggest these conserved amino acids play a role in substrate selectivity for Dop.

*Mycobacterium tuberculosis* is a human exclusive pathogen that is transmitted by aerosols and causes the disease tuberculosis (TB). Although TB can be effectively treated with several antibiotics, treatment is prolonged, which often results in poor compliance and the emergence of drug-resistant strains. In an effort to find new targets for TB treatment, a screen for mutants sensitive to the host effector nitric oxide (NO) identified mutations in components of the bacterial proteasome system (1). In eukaryotes, proteins targeted for proteasomal degradation are posttranslationally modified by the small protein ubiquitin (reviewed in (2)), whereas bacteria have a different modification called Pup. In *M. tuberculosis*, Pup is translated as a 64 amino acid protein ending in glutamine (Gln) that must be deamidated to glutamate (Glu) by deamidase of Pup (Dop) prior to attachment by the only known Pup ligase, proteasome accessory factor A (PafA), to substrate lysines (3, 4, 5). The pupylation status of any protein is likely dynamic given that Dop can also remove Pup from substrates (depupylation), rescuing them from degradation (6, 7), and PafA can potentially move Pup from one substrate to another (8). Given that over 60 proteins are targets of pupylation that comprise the “pupylome” (9, 10, 11), it is perhaps unsurprising that components of the Pup-proteasome system (PPS) are essential for the robust virulence of *M. tuberculosis* in animal models (1, 12, 13, 14). In fact, the accumulation of a single proteasome substrate, Log, results in a buildup of aldehydes that synergize with NO to kill bacteria and attenuate growth in mice, demonstrating the essential robustness of the PPS for resistance to host defenses and potentially other stressors (15).

A major gap in understanding the PPS is how proteins are selected for pupylation and depupylation. The expression of *M. tuberculosis dop*, *pup*, and *pafA* in *Escherichia coli*, which lacks a PPS, results in the pupylation of numerous proteins (16), suggesting that there is no mycobacteria-specific sequence motif that PafA must recognize to pupylate a protein. PafA and Dop are members of the glutamine synthetase superfamily and share numerous conserved residues in their active sites (13, 17, 18). While PafA catalyzes a reaction similar to glutamine synthetases, Dop does not. Dop has an amidase activity that appears unique to it and its close homologs (17, 19, 20). Furthermore, Dop has a disordered loop sequence that is absent in PafA and is a highly conserved region among Dops from diverse actinobacterial species (17). Deletion of the "Dop loop" does not diminish its activity nor does it convert Dop into a ligase. However, deletion of the loop and addition of an alpha helix from PafA confers ligase activity to *Mycobacterium smegmatis* Dop (21).

In a study by the Gur lab, *in vitro* analysis found that deletions in the *M. smegmatis* Dop loop result in enzymes that more rapidly depupylate model substrates. Steady state pupylomes in *M. smegmatis* expressing mutant *dop* are reduced compared to the pupylome from a strain expressing wild-type (wt) *dop*, suggesting these Dop loop mutant alleles also hyperdepupylate *in vivo* (22). The authors of this work also showed that Dop binding to one substrate, Pup∼IdeR, is unaffected by the Dop loop deletion, concluding the Dop loop regulates catalysis and not substrate binding. In contrast, the Weber-Ban lab found that replacement of loop residues with different amino acids made *Corynebacterium glutamicum* Dop more slowly depupylate a model substrate. Moreover, the authors proposed that the Dop loop promotes the dephosphorylation of an active site nucleotide (ATP), releasing a phosphate needed for amidase activity (20). It is possible that differences in Dop loop function described in these studies were in part due to the use of Dop from different species (*M. smegmatis* Dop is 50% identical/75% similar to *C. glutamicum* Dop).

We sought to understand how this highly conserved and unstructured region of Dop affects the proteome of *M. tuberculosis*. We complemented a *dop* transposon mutation with either integrative or overexpression plasmids encoding various *dop* alleles, including a large deletion encompassing most of the conserved amino acids or several smaller deletions within the loop, and assessed the pupylomes of these strains. Deletion of the Dop loop resulted in an overall reduced pupylome and the accumulation of several established proteasome substrates, supporting observations in *M. smegmatis* (22). Smaller deletions of the Dop loop had variable effects, affecting only a handful of established PPS substrates. Most interestingly, the overexpression of mutant *dop* loop alleles resulted in dramatically different pupylomes. In particular, the expression of a specific *dop* loop deletion allele resulted in the accumulation of a single pupylated protein, suggesting the deleted amino acids are important for depupylating this substrate. Collectively, we propose that residues in the Dop loop help regulate depupylation, possibly by affecting access to substrates.

## Results

### Deletion of amino acids in the conserved Dop loop reduced pupylome abundance

**Figure 1:**
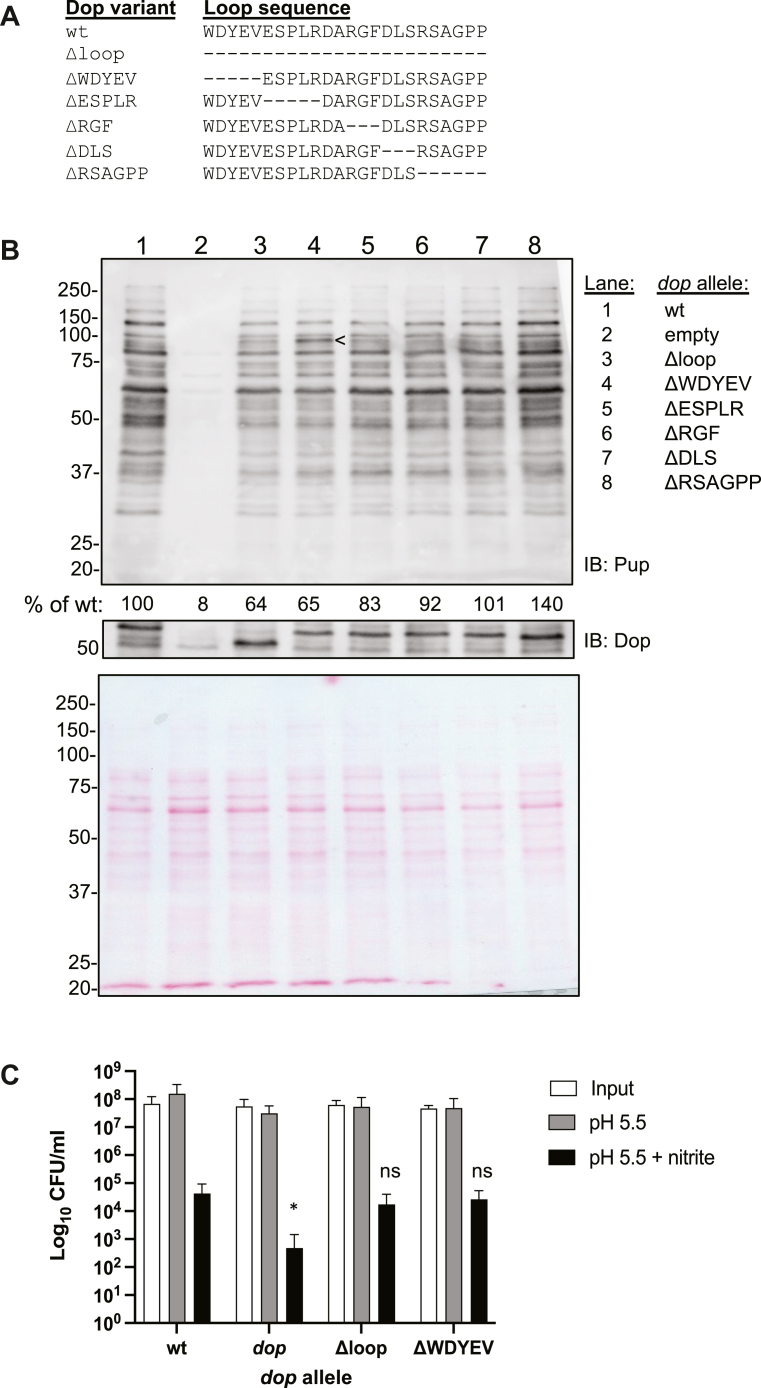
**Amino acid deletions in the Dop-loop affected pupylation levels in *M. tuberculosis*.***A*, amino acids deleted from the Dop loop region. In *M. tuberculosis*, these residues represent amino acids 48 to 71. *B*, an *M. tuberculosis dop*-null strain was complemented with integrative plasmids encoding *dop* with deletions in the Dop loop. Equivalent bacterial cell numbers were harvested and lysed for analysis on a 10% SDS-PAGE gel by immunoblotting (IB) for Pup. The pupylomes were quantified using Fiji and compared to the amount in the wt *dop* complemented strain. Arrowhead (<) indicates a unique species accumulating in the ΔWDYEV strain. Dop levels were checked by stripping the same membrane and incubating with antibodies to Dop. Molecular weight (MW) standards in kD are indicated on the left. Ponceau S-stained membrane before IB is shown at the bottom as a loading control. *C*, loop mutations did not affect NO sensitivity. The first four strains in (*B*) were incubated for 6 days in acidified media with or without 3 mM nitrite and then plated on agar to enumerate surviving colony forming units (CFU) 2 to 3 weeks later. Data are representative of three independent experiments, each performed in triplicate, with error bars signifying means ± standard deviation (SD). Statistical analysis was done by performing unpaired *t* tests comparing mutant strains to WT *dop-*complemented strain. ∗*p* < 0.05; ns = not statistically significant.

In *M. smegmatis*, Dop lacking the loop depupylates faster than wt Dop *in vitro* and *in vivo*, suggesting that the Dop loop inhibits depupylation (22). To test if deletion of the loop would have a similar effect in *M. tuberculosis*, we complemented an *M. tuberculosis dop* transposon mutation with an integrative plasmid encoding various deletions from the *dop* loop sequence; *dop* alleles were expressed from the native *dop* promoter (see Table 1). We deleted the coding sequence for the 24 most conserved amino acids (“Δloop”) as well as made shorter deletions within the loop (Fig. 1*A*) and assessed pupylome levels at steady state by immunoblotting (Fig. 1*B*). As previously reported in *M. tuberculosis*, complementation of this *dop* mutant with WT *dop* restores a robust pupylome (Fig. 1*B*, lanes 1 *versus* 2) (13). The strain complemented with Δloop had a reduced pupylome (Fig. 1*B*, lane 3), similar to what was previously observed in *M. smegmatis* producing Dop lacking either 14 or 37 residues from its loop (22).

**Table 1:** Bacterial strains, plasmids, and primers used in this work

*E. coli*:	Relevant genotype:	Source or reference:
DH5α	F-, θ80Δ*lac*ZM15 Δ(*lacZYA-argF*)U169 *deoR recA1 endA1* *hsdR17* (r_k_-m_k_+) *phoA supE44* λ- *thi-1 gyrA96 relA1*	Gibco, BRL.
***M. tuberculosis*:**		
CDC1551	wild type	W. Bishai collection
MHD58 (MT2172)	CDC1551 *dop*::MycoMarT7; Kan^r^	(13)
MHD375	MHD58 pMV306; Hyg^r^, Kan^r^	(13)
MHD376	MHD58 pMV-*dop*; Hyg^r^, Kan^r^	(13)
MHD1628	MHD58 pMV-*dop*_Δloop_; Hyg^r^, Kan^r^	This work.
MHD1631	MHD58 pMV-*dop*_ΔWDYEV_; Hyg^r^, Kan^r^	This work.
MHD1632	MHD58 pMV-*dop*_ΔESPLR_; Hyg^r^, Kan^r^	This work.
MHD1630	MHD58 pMV*-dop*_ΔRGF_; Hyg^r^, Kan^r^	This work.
MHD1633	MHD58 pMV*-dop*_ΔDLS_; Hyg^r^, Kan^r^	This work.
MHD1629	MHD58 pMV-*dop*_ΔRSAGPP._; Hyg^r^, Kan^r^	This work.
MHD671	MHD58 pOLYG; Hyg^r^	This work.
MHD1097	MHD 58 pOLYG-*dop*; TAP-tagged; Hyg^r^, Kan^r^	This work.
MHD1663	MHD 58 pOLYG-dop_Δloop_; TAP-tagged; Hyg^r^, Kan^r^	This work.
MHD1664	MHD 58 pOLYG-*dop*_ΔWDYEV_; TAP-tagged; Hyg^r^, Kan^r^	This work.
MHD1681	MHD 58 pOLYG-*dop*_ΔESPLR_; TAP-tagged; Hyg^r^, Kan^r^	This work.
MHD1682	MHD 58 pOLYG-*dop*_ΔRGF_; TAP-tagged; Hyg^r^, Kan^r^	This work.
MHD1683	MHD 58 pOLYG-*dop*_ΔDLS_; TAP-tagged; Hyg^r^, Kan^r^	This work.
MHD1684	MHD 58 pOLYG-*dop*_ΔRSAGPP_; TAP-tagged; Hyg^r^, Kan^r^	This work.
Δ*nuoAN*	CDC1551 with a deletion of *nuoA* through *nuoN*	(23)
MHD1701	Δ*nuoAN* pOLYG; Hyg^r^	This work.
MHD1702	Δ*nuoAN* pOLYG-*dop*; TAP-tagged; Hyg^r^	This work.
MHD1703	Δ*nuoAN* pOLYG-*dop*_ΔWDYEV_; TAP-tagged; Hyg^r^	This work.

Plasmids	Description	Reference

pOLYG	Hyg^r^; shuttle plasmid for gene overexpression in mycobacteria	(36)
pMV306	Hyg^r^; mycobacterial plasmid that integrates at *attB* site on mycobacterial chromosomes	(37)


Primers (sequences are 5′ to 3′):	
Primers	Sequence (5′ to 3′)
pOLYGfor	CATGACCAACTTCGATAACG
pOLYGrev	GCACGACAGGTTTCCCGACTG
dopTAP_loop-WDYEV_R	GCGCAGCGGCGATTCACGGGTGCGTTTGGC
dopTAP_loop-WDYEV_F	GCCAAACGCACCCGTGAATCGCCGCTGCGC
dopTAP_loop-ESPLR_R	GAAGCCCCGGGCGTCCACCTCGTAGTCCCA
dopTAP_loop-ESPLR_F	TGGGACTACGAGGTGGACGCCCGGGGCTTC
dopTAP_loop-DA_R	CAAATCGAAGCCCCGGCGCAGCGGCGATTC
dopTAP_loop-DA_F	GAATCGCCGCTGCGCCGGGGCTTCGATTTG
dopTAP_loop-RGF_R	CGAGCGACTCAAATCGGCGTCGCGCAGCGGCGATTCCACC
dopTAP_loop-RGF_F	GGTGGAATCGCCGCTGCGCGACGCCGATTTGAGTCGCTCG
dopTAP_loop-DLS_R	CGGCCCGGCCGAGCGGAAGCCCCGGGCGTCGCGCAGCGGC
dopTAP_loop-DLS_F	GCCGCTGCGCGACGCCCGGGGCTTCCGCTCGGCCGGGCCG
dopTAP_loop-RSAGPP_R	GGCGTCGACCACCGGACTCAAATCGAAGCC
dopTAP_loop-RSAGPP_F	GGCTTCGATTTGAGTCCGGTGGTCGACGCC
Dop_24cleandel_F	AGCGTGCCAAACGCACCCGTCCGGTGGTCGACGCCGACGA
Dop_24cleandel_R	TCGTCGGCGTCGACCACCGGACGGGTGCGTTTGGCACGCT
pMV306for	CGGTTCCTGGCCTTTTGCTGGCC
pMV306seqR	CCTGTCGTTCACGGCTCTA

The smaller amino acid deletions in the Dop loop also resulted in decreased pupylome abundance. Deletions nearer to the amino terminus had greater decreases in pupylome levels; the strain producing Dop lacking the amino acids tryptophan, aspartate, tyrosine, glutamate, and valine (“ΔWDYEV”) had the most similar pupylome to the Δloop strain (Fig. 1*B*, lanes 3 *versus* 4). This decrease in pupylome abundance was specifically due to the deleted residues and not just the shortening of the Dop loop, given that deletion of six residues (arginine, serine, alanine, glycine, proline, proline; ΔRSAGPP) at the carboxyl terminus of the loop resulted in a pupylome like the wt-complemented strain (Fig. 1*B*, lanes 1 *versus* 8).

Deletion of the loop from *M. smegmatis* Dop does not affect deamidation activity (22). Thus, it seemed unlikely that the decreases in pupylome levels seen in Figure 1 were due to the reduced conversion of newly translated Pup_Gln_ to Pup_Glu_. Instead, we hypothesized that the reduced pupylome levels were due to either slower or faster depupylation by the various Dop alleles. Hypodepupylation would result in more protein getting targeted to the proteasome, thus reducing the abundance of known proteasome substrates. In contrast, hyperdepupylation could rescue these substrates from degradation, thereby increasing the amount of a substrate relative to its abundance in wt bacteria. To determine which of these scenarios was more likely, we quantified and compared the proteome of the *dop*-null mutant to the proteomes of strains producing wt, Δloop, and ΔWDYEV Dop using tandem-mass tag mass spectrometry (TMT-MS). As expected, the *dop*-null mutant had the highest accumulation of several established proteasome substrates given that there is no pupylation in this strain (Tables 2, and S1). In the strains producing Δloop or ΔWDYEV alleles, several proteasome substrates accumulated but to a lesser degree than what were observed in the *dop*-null strain (Tables 2 and S1). Nonetheless, this result suggested these mutant loop Dop alleles hyperdepupylated several known proteasome substrates, rescuing them from proteasomal degradation.

**Table 2:** Mutations in the Dop loop resulted in increased levels of a subset of pupylated substrates. "+" indicates the protein was statistically significantly more abundant in the respective strain compared to a strain producing wt Dop

Substrate:	MW (kD):	Dop null	Δloop	ΔWDYEV
FabD	31	+	+	+
KasA	43	+	+	+
Icl	47	+	+	+
Log	20	+	+	
PanB	29	+	+	
Ino1	40	+	+	
FusA	77	+	+	
Bcp	17	+		
LeuD	22	+		
MtrA	25	+		
NuoE	27	+		
Rv2859c	32	+		
Rv0073	36	+		
FadA	42	+		
MurA	44	+		
PhoH2	47	+		
PafA	50	+		
GlmU	52	+		
SahH	54	+		
Mpa	67	+		
RecA	85	+		

See Table S1 for full list of quantified proteins.

Abbreviation: MW, molecular weight.

Defective protein degradation by proteasomes is associated with an increased susceptibility of *M. tuberculosis* to NO due to the failed degradation of the proteasome substrate Log (15). Log did not accumulate in any of the tested loop mutants (Table S1), but we nonetheless tested whether or not the small loop deletions affected NO susceptibility. Consistent with our observation that Log did not accumulate in any of the tested loop mutant strains, none of these strains was hypersensitive to NO (Fig. 1*C*).

The decrease in pupylome levels was unlikely due to changes in the abundance of the proteasome subunits (PrcA and PrcB) and mycobacterial proteasome activator Mpa because they were present at similar levels in the analyzed strains (Table S1). In contrast, there was less Pup in the mutant strains relative to the strain making wt Dop (Table S1). Pup is highly unstable when not conjugated to another protein in *M. tuberculosis* (13). Thus, the reduced Pup levels in the Dop loop mutants, along with the accumulation of known proteasome substrates, is consistent with a model in which hyperdepupylation occurs in these bacteria. However, we could not rule out an alternative explanation in which Dop loop mutations negatively influenced the ability of Dop to depupylate certain substrates, an activity that could also affect the overall Pup pool.

### Overexpression of loop mutant alleles revealed variable pupylomes

**Figure 2:**
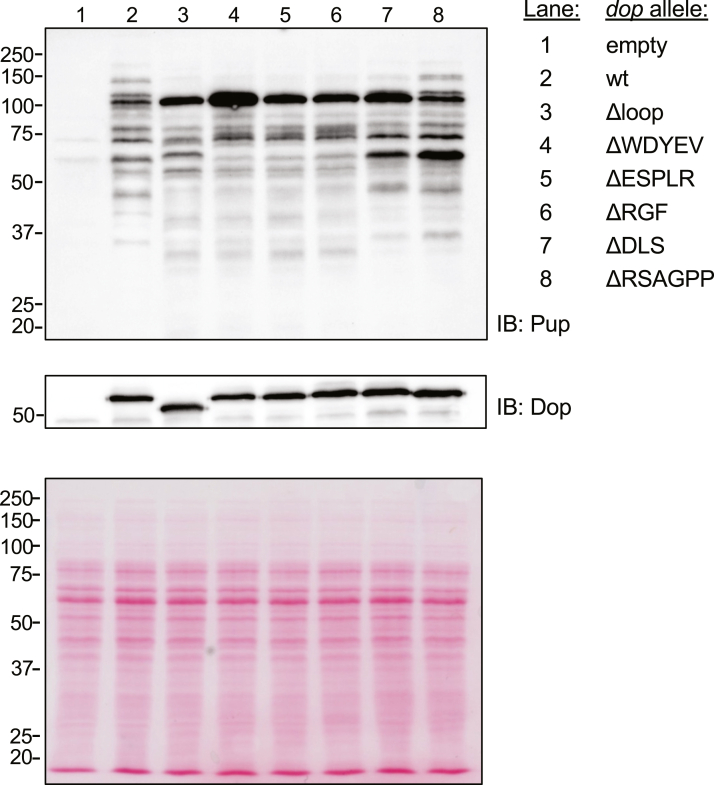
**Overproduction ofDop loop variants resulted in variable pupylomes.** An *M. tuberculosis dop*-null strain was transformed with an overexpression plasmid encoding various deletions in the *dop* loop. Equivalent cell numbers were harvested for lysis, and lysates were separated by 10% SDS-PAGE. Pupylated proteins were analyzed by IB for Pup. The same blot was stripped and incubated with antibodies to Dop to check relative Dop levels among the strains. As a loading control, Ponceau S-stained membrane is shown at the bottom. MW standards are indicated on the left. IB, immunoblotting; MW, molecular weight in kD.

While the relative amounts of pupylated protein varied, the banding pattern of the pupylomes in our immunoblots did not appear different among the strains expressing the various loop alleles (Fig. 1*B*). However, an accumulated species of about 100 kD was apparent in the strain producing the ΔWDYEV allele (Fig. 1*B*, lane 4, arrowhead). Based on this observation, we hypothesized that specific residues in the Dop loop contributed to the depupylation of certain proteins. To begin to test this hypothesis, we overexpressed wt *dop* and mutant loop alleles in the *dop*-null *M. tuberculosis* strain, with the expectation that overexpression might magnify differences among the Dop alleles. We performed immunoblot analysis on total cell lysates of these strains and observed that several of the mutant *dop* allele-expressing strains had distinct pupylomes, with multiple pupylated proteins that were more prominent in several strains compared to each other or the WT *dop-*expressing strain (Fig. 2, lane 2 *versus* lanes 3–8).

**Figure 3:**
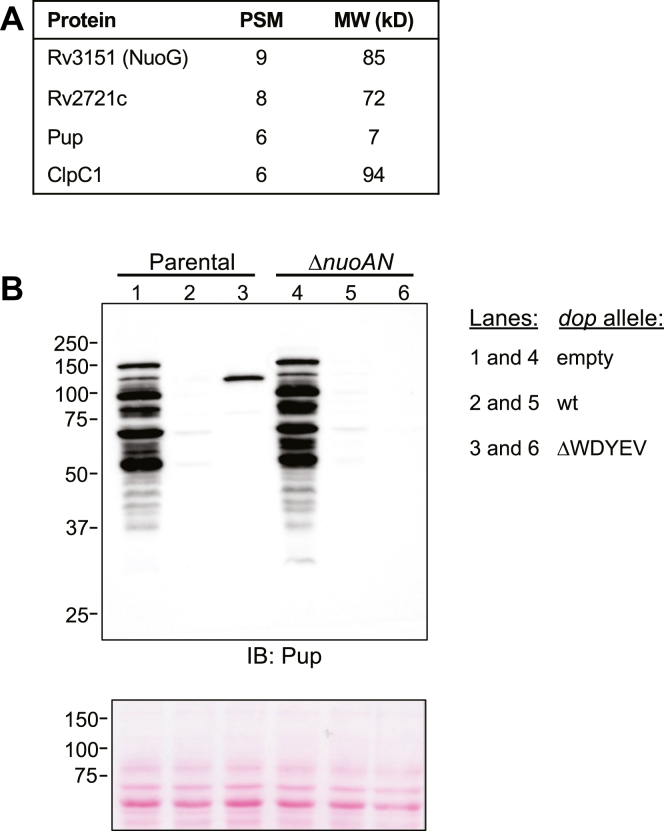
**NuoG was hyperpupylated in *M. tuberculosis* overproducingDopΔWDYEV.***A*, top peptide-spectrum match (PSM) hits identified from the Pup immunoprecipitations. *B*, whole cell lysates were collected from equivalent amounts of bacteria and separated by 10% SDS PAGE. Pupylated proteins were analyzed by IB for Pup. Ponceau S-stained membrane before IB is shown at the *bottom* as a loading control. MW standards are indicated on the left. IB, immunoblotting; MW, molecular weight in kD.

In most of the loop mutants, an approximately 100 kD species, herein called “protein X,” was present at greater levels than in the wt *dop*-expressing strain and most dramatically accumulated in the ΔWDYEV strain (Fig. 2, lane 4); it was likely that protein X was the same species seen in Figure 1*B*, lane 4. We hypothesized that the identity of protein X could give some insight into the significance of the WDYEV sequence in the Dop loop. To identify protein X, we performed immunoprecipitations using mAbs to Pup. After separating immunoprecipitated proteins by SDS-PAGE, we excised the region around 100 kD for MS analysis. After tryptic digestion and MS analysis, the top proteins with more than five peptide spectral matches included Pup and NuoG (Fig. 3*A*).

NuoG is an 85 kD protein and part of the 14-subunit type 1 NADH dehydrogenase complex that is encoded by the *nuoA* operon (23). To further test if NuoG was indeed protein X, we tested for protein X accumulation in a Δ*nuoAN* mutant lacking the entire operon and overexpressing wt or ΔWDYEV *dop* alleles. Robust pupylomes were seen in both the parental and Δ*nuoAN* strains when transformed with empty vector (Fig. 3*B*, lanes 1 and 4), whereas the overexpression of wt *dop* resulted in dramatically reduced pupylomes (Fig. 3*B*, lanes 2 and 5), most likely due to hyperdepupylation. Nonetheless, the overproduction of the ΔWDYEV mutant resulted in the appearance of protein X in the parental strain as seen in Figure 2 but not in the Δ*nuoAN* strain. Because none of the other proteins encoded in the *nuoA* operon was identified by our proteomics analysis and all of the Nuo proteins except NuoG are 66 kD or smaller, we concluded that protein X is Pup∼NuoG.

**Figure 4:**
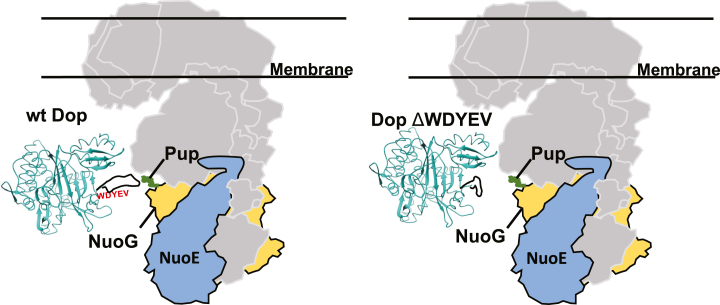
**Location of NuoG within a complex may affect its depupylation by the ΔWDYEV Dop mutant.** NuoG (*yellow*) is part of the type I NADH dehydrogenase complex that includes the proteasome substrate NuoE (*blue*). Pup was placed at an arbitrary location on NuoG. Chimera (32) was used to model *Acidothermus cellulolyticus* Dop (*light sea green*) from PDB 4B0R with its disordered loop (*black line*) manually added. The active site of Dop is in the β-sheet cradle. NADH complex model is based on the proposed assembly of the complex in *E. coli* (24). PDB, Protein Data Bank.

NuoG is a part of the peripheral arm of the type 1 NADH dehydrogenase complex (24) and has never been identified as a proteasome substrate in *M. tuberculosis*. Under routine culture conditions used in this work, we did not observe an accumulation of NuoG in the *dop* mutant, which we would expect if NuoG were a proteasome substrate (Table S1). In contrast, NuoE, which is also a part of this complex, is a confirmed pupylated substrate that accumulated in the *dop*-null mutant (Table 2) (9). Although we do not know which lysine in NuoG is pupylated, it is possible that access to this residue is affected by its location within the NADH dehydrogenase complex (Fig. 4).

## Discussion

In this study, we sought to understand the *in vivo* function of a highly conserved loop sequence in the *M. tuberculosis* amidase Dop. We showed that the effect of the loop deletions depended on which residues were deleted, and deletion of as few as three amino acids from the Dop loop had global effects on pupylome levels. The overexpression of a specific *dop* allele, ΔWDYEV, resulted in the dramatic accumulation of Pup∼NuoG, suggesting this substrate could not be efficiently depupylated by this mutant Dop. Thus, our data suggest highly conserved amino acids in the Dop loop regulate the ability of Dop to depupylate certain substrates in *M. tuberculosis*.

Previous work by two other groups worked to understand the function of the Dop loop. Both studies concluded that the loop affected the rate of catalysis by Dop but in contradictory ways (20, 22). In one study, deletion of the entire loop sequence or replacement of seven highly conserved residues in the loop with glycine or serine resulted in faster depupylation of three model substrates *in vitro* (22). In another study, deletion of the Dop loop resulted in slightly slower depupylation of a model substrate and a fluorescent probe (20). It is possible that the differences observed by the two groups were due to the use of Dop from different bacterial species.

It was also proposed that the highly conserved tryptophan of the WDYEV sequence in the Dop loop stabilizes ATP binding and hydrolysis, with the liberation of phosphate suggested to be required for depupylation (20). However, deletion of the loop, which includes WDYEV, does not abolish Dop activity as reported here and elsewhere (17, 22). Perhaps most relevantly, ATP hydrolysis is not required *per se* for depupylation given that ADP and Pi are sufficient for Dop to robustly catalyze depupylation *in vitro* (8, 25). Thus, it seems unlikely that the loop plays a role in nucleotide hydrolysis to promote depupylation.

Our *M. tuberculosis* results support a scenario observed in *M. smegmatis* in which the loop plays an inhibitory role in depupylation. However, this does not appear to be the only effect of the Dop loop on the pupylome. In addition to a model where hyperdepupylation by Dop loop mutants led to a reduced pupylome, it is possible that proteins were underpupylated due to a reduction in the overall Pup pool. This idea is based on the observation that Pup recycling by Dop is essential to maintain a robust pupylome in *M. tuberculosis*; if Pup is not constantly removed and reattached to substrates, the pupylome is substantially diminished (13). Thus, it is possible that if proteins like NuoG are inefficiently depupylated, the overall Pup pool would be insufficient to maintain a wt pupylome. Taken together, it is possible the loop can both restrict and promote depupylation depending on the substrate.

Another hypothesis we propose is that some pupylated proteins act as sources of Pup to be directly transferred from one substrate to another (8). This hypothesis arose from the observation that all enzymes encoded in the fatty acid synthase II (FASII) biosynthetic pathway operon are pupylated, but only FabD is robustly degraded under steady state conditions (3, 9, 10, 26). Given that PafA can transfer Pup from one substrate to another *in vitro* (8), it is possible PafA can transfer Pup from one or more of the other pupylated FASII enzymes to FabD to facilitate its degradation. Although NuoE did not accumulate in the ΔWDYEV strain, it is still possible that Pup∼NuoG is a source of Pup to promote the degradation of NuoE or other nearby proteasome substrates under different conditions.

The selection mechanisms of proteins to be pupylated or depupylated remain to be determined. In particular, how do loop residues affect depupylation? It is notable that Pup∼NuoG did not accumulate as dramatically in the Δloop strain, which lacks the WDYEV sequence, as it did in the ΔWDYEV strain. This result suggests that deletion of WDYEV may cause a conformation in Dop that prevents active site access to the isopeptide bond in Pup∼NuoG, a block that is alleviated when more loop sequence is deleted. Thus, the conserved loop sequence may have evolved to affect how Dop accesses certain types of pupylated substrates. Another possibility is that the loop affects interactions with other yet-to-be-identified proteins that facilitate the depupylation of some substrates. While the loop is predicted to be unstructured, intrinsically disordered proteins (IDPs) frequently achieve structure when interacting with a specific binding partner (reviewed in (27)). A relevant example in *M. tuberculosis* is the interaction of Pup, an IDP, with Mpa, the receptor and chaperone of Pup-dependent proteasomal degradation. In solution, Pup is mostly disordered but forms a robust interaction with the N termini of Mpa in a hexamer (28, 29, 30). Thus, it remains to be determined if the Dop loop directly interacts with substrates or binds to one or more proteins that facilitate depupylation.

## Experimental procedures

### Strains, plasmids, primers, and culture conditions

See Table 1 for strains, plasmids, and primers used in this work. Reagents used for making all buffers and bacterial media were purchased from Thermo Fisher Scientific, unless otherwise indicated. *M. tuberculosis* was grown in "7H9c" (BD Difco Middlebrook 7H9 broth with 0.2% glycerol and supplemented with 0.5% bovine serum albumin, 0.2% dextrose, 0.085% sodium chloride, and 0.05% Tween-80). For solid media, *M. tuberculosis* was grown on Middlebrook 7H11 agar (“7H11”, BD Difco) containing 0.5% glycerol and supplemented with 10% final volume of BBL Middlebrook OADC Enrichment. For selection of *M. tuberculosis*, the following antibiotics were used as needed: kanamycin 50 μg/ml and hygromycin 50 μg/ml. *E. coli* was cultured in Luria-Bertani broth or on Luria-Bertani agar (both BD Difco). Media were supplemented with the following antibiotics as needed: kanamycin 100 μg/ml and hygromycin 150 μg/ml.

Dop loop deletions were made by splicing overlap extension PCR with Phusion polymerase (31). *dop* encoding C-terminal hexahistidine and FLAG tags were cloned into the BamHI and HindIII sites of pOLYG for overexpression. To make integrative plasmids with the same mutant sequences, we used the overexpression plasmids as templates for PCR to add HindIII and XbaI cut sites and remove the affinity tag sequences. PCR products were cloned into plasmid pMV306. Calcium chloride–competent *E. coli* DH5α was used for transformations. Plasmids were purified from *E. coli* using the QIAprep Spin Miniprep Kit (Qiagen). All plasmids made by PCR cloning were sequenced by GENEWIZ, Inc to ensure the veracity of the cloned sequence. Primers used for PCR amplification or sequencing were purchased from Life Technologies and are listed in Table 1. DNA was PCR amplified using Phusion polymerase (New England Biolabs; NEB) according to the manufacturer’s instructions. PCR products were purified using the QIAquick Gel Extraction Kit (Qiagen). Restriction enzymes and T4 DNA ligase were purchased from NEB.

*M. tuberculosis* was transformed by electroporation as previously described (32). All *M. tuberculosis* work was performed in the ABSL3 facility of NYU Grossman School of Medicine, in accordance with its Biosafety Manual and Standard Operating Procedures.

### Preparation of *M. tuberculosis* extracts for immunoblotting

*M. tuberculosis* cultures were grown to an absorbance at 580 nm (*A*_580_) of ∼1. Equivalent cell numbers were collected based on the *A*_580_ of the cultures. For example, an “*A*_580_ equivalent of 1” indicates the *A*_580_ of a 1 ml culture is 1.0. Five *A*_580_ equivalents of bacteria were harvested by centrifugation at 3000*g*, washed in PBST (PBS, 0.05% Tween 80), resuspended in lysis buffer (100 mM Tris-Cl pH8, 1 mM EDTA pH8), and transferred to a tube containing 250 μl of 0.1 mm zirconia beads (BioSpec Products). Bacteria were lysed using a mechanical bead-beater (BioSpec Products). Whole-cell lysates were mixed with 4× reducing SDS sample buffer (250 mM Tris pH 6.8, 2% SDS, 20% 2-mercaptoethanol, 40% glycerol, 1% bromophenol blue) to a 1× final concentration, and samples were boiled for 10 min at 100 °C.

For immunoblotting, whole cell lysates were separated by 10% SDS-PAGE. Proteins were transferred to nitrocellulose membranes (GE Amersham) by semidry transfer and blocked with 3% bovine serum albumin or 2% milk in 0.1× Tris-buffered saline with Tween-20 (TBST); use of 0.1× TBST allowed for a more sensitive detection of the pupylome. Membranes were incubated with monoclonal Pup antibody reported previously (13). Horseradish peroxidase–conjugated secondary antibodies to mouse and rabbit IgG were purchased from Pierce. Immunoblots were developed using SuperSignal West Pico PLUS chemiluminescent substrate (Thermo Fisher Scientific) and imaged using Bio-Rad ChemiDoc system and quantified using Fiji (33). Blots were stripped as previously described (34). The membrane was reblocked with 2% milk in 1× TBST, incubated with polyclonal Dop antibodies reported previously (6), and imaged as aforementioned.

### TMT-MS

*M. tuberculosis* expressing wt *dop*, Δloop, and ΔWDYEV were grown as described previously. To prepare samples for TMT-MS, *M. tuberculosis* strains were grown to an *A*_580_ of 1 to 1.3. Nineteen *A*_580_ equivalents of bacteria were collected as stated previously. Insoluble debris was pelleted by centrifugation for 1 min at top speed in a microfuge. Lysates were filter sterilized using 0.2 μm nylon Spin-X columns (Costar). Sterilized samples were submitted to the NYUMC Proteomics Laboratory for proteome quantification by TMT-MS.

Samples were reduced using DTT for 1 h at 55  °C and reduced cysteines were alkylated with iodoacetamide. Each sample was loaded onto S-Trap microcolumns (ProtiFi) according to the manufacturer’s instructions. Samples were centrifuged at 4000*g* for 30 s. After three washes, proteins were trypsinized and peptides were eluted with 40% acetonitrile (ACN) in 0.5% acetic acid followed by 80% ACN in 0.5% acetic acid. Eluted peptides were dried and concentrated in a SpeedVac. The dried peptide mixture was resuspended in 100 mM TEAB (pH 8.5). Each sample was labeled with TMT reagent according to the manufacturer’s protocol. The samples were then combined at a 1:1 ratio, and the pooled sample was subsequently desalted using C18 solid-phase extraction (Harvard Apparatus). Aliquots of pooled samples were fractionated using a 4.6 mm × 250 mm Xbridge C18 column (Waters, 3.5 μm bead size) with an Agilent 1260 Infinity Bio-inert HPLC and separated over a 70 min linear gradient from 10% to 50% solvent B at a flow rate of 0.5 ml/min (Buffer A = 10 mM ammonium formate, pH 10.0; Buffer B = 90% ACN, 10 mM ammonium formate, pH 10.0). A total of 45 fractions were collected throughout the gradient. The early, middle, and late eluting fractions were concatenated and combined into 15 final fractions. The combined fractions were concentrated in the SpeedVac and stored at −80 °C until further analysis.

An aliquot of each sample was loaded onto a trap column (Acclaim PepMap 100 precolumn, 75 μm × 2 cm, C18, 3 μm, 100 Å, Thermo Scientific) connected to an analytical column (EASY-Spray column, 50 m × 75 μm ID, PepMap RSLC C18, 2 μm, 100 Å, Thermo Scientific) using the autosampler of an Easy nLC 1200 (Thermo Scientific) with solvent A consisting of 2% ACN in 0.5% acetic acid and solvent B consisting of 80% ACN in 0.5% acetic acid. The peptide mixture was gradient eluted into the Orbitrap Eclipse Tribrid mass spectrometer (Thermo Scientific) using the following gradient: a 5% to 15% solvent B in 60 min, 15% to 25% solvent B in 45 min, 25% to 40% solvent B in 15 min, followed by 40% to 100% solvent B in 20 min. High resolution full MS spectra were obtained with a resolution of 60,000 (@*m*/*z* 200), an automatic gain control target of 4e5, with a maximum ion time of 50 ms, and a scan range from 400 to 1500 m/z. Following each full MS scan, high resolution MS/MS spectra were acquired for a 3 s duty cycle using the following parameters: resolution 60,000 (@*m*/*z* 200), isolation window of 0.7 *m*/*z*, target value of 1e5, maximum ion time of 60 ms, normalized collision energy of 30, and dynamic exclusion of 30 s.

MS data were analyzed using MaxQuant software version 1.6.15.0 (https://www.maxquant.org/) (35) and searched against the *M. tuberculosis* H37Rv proteome, using the following settings: oxidized methionine (M) and deamidation (NQ) were selected as variable modifications and carbamidomethyl (C) as fixed modifications; false discovery rate for peptide, protein, and site identification was set to 1% and was calculated using a decoy database approach. The minimum peptide length was set to 6. The following filters and criteria were used for quantification: proteins identified with less than two unique peptides were excluded from analysis. Bioinformatics analysis was performed with Perseus and R Studio. Student’s *t* test using a 0.05 *p*-value cutoff was then used to identify proteins that were differentially expressed. The values in Table 2 were calculated by taking the inverse log of the ratios of proteins in loop mutant to wt Dop expressing strains.

### Nitric oxide sensitivity assay

Assays were performed as described previously (1). Briefly, bacteria were grown to an *A*_580_ ∼0.8 to 1 and resuspended in acidified 7H9c (pH 5.5) and diluted to an *A*_580_ of 0.08. Bacteria were then aliquoted in triplicate to flat bottom 96-well plates, and a fresh sodium nitrite solution was added to each well at a final concentration of 3 mM. Bacteria were incubated for 6 days at 37 °C before plating onto 7H11 OADC plates and incubated at 37 °C for enumeration 2 to 3 weeks later.

### Immunoprecipitations

To identify protein X, nProtein A Sepharose Fastflow beads (GE Healthcare) were incubated with Pup mAb rotating for 3 h at 4 °C. *Mtb dop* null strain with pOLYG-*dop*ΔWDYEV was grown to *A*_580_ of 1.6 and 30 *A*_580_ equivalents were harvested by centrifugation. The pellet was washed with lysis buffer (PBS with DNAseI, cOmplete Mini EDTA free protease inhibitor tablets (Roche)), transferred to a tube with 200 μl zirconia beads, and bead beat for 3 × 30 s. The lysates were centrifuged for 10 min 10,000*g* at 4 °C and then filtered with 0.22 μM cellulose Spin-X filters by centrifugation for 5 min at the same settings. The lysates were precleared to minimize nonspecific binding proteins by incubating them with the protein A-Sepharose slurry for 1 h at 4 °C. The beads were centrifuged to pellet and the lysate was transferred to a new tube with 50 μl of ProteinA Sepharose beads and incubated overnight. Unbound protein was removed by centrifugation and then beads were washed with 1× PBS. The beads were resuspended in 50 μl 2 × SDS sample buffer and boiled for 10 min. The proteins in the eluate were separated by SDS-PAGE and the gel stained with Coomassie brilliant blue. We excised a region of the gel just below and above 100 kD marker and submitted the sample to the NYUMC Proteomics Laboratory for identification.

## Data availability

All data supporting these findings are available within the article and/or its supplementary materials.

## Supporting information

This article contains supporting information Table S1.

## Conflicts of interest

The authors declare that they have no conflicts of interest with the contents of this article.

## Supporting information

TableS1
